# Coupling Photothermal Effect in N-Doped Hollow Carbon Spheres with ZnIn_2_S_4_ Boosts Solar Hydrogen Evolution

**DOI:** 10.3390/molecules30224368

**Published:** 2025-11-12

**Authors:** Shanhao He, Li Liu, Min Liu, Jinjun Tian, Yan Xue, Keliang Wu

**Affiliations:** 1 School of Biology and Chemical Engineering/Henan Key Laboratory of Microbialfermentation, Nanyang Institute of Technology, Nanyang 473000, China; 2 School of Chemistry and Chemical Engineering/State Key Laboratory Incubation Base for Green Processing of Chemical Engineering, Shihezi University, Shihezi 832003, China; 3 School of Petrochemical Engineering, Bayingolin Vocational and Technical College, Korla 841000, China

**Keywords:** Zinc Indium Sulfide, N-doped hollow carbon spheres, photothermal effect, hydrogen evolution

## Abstract

To address the challenges of low solar energy utilization efficiency and rapid recombination of photogenerated charge carriers in photocatalytic hydrogen evolution, this study successfully constructed a composite photocatalyst of ZnIn_2_S_4_ (ZIS) supported on N-doped hollow carbon spheres (N-HCS), denoted as ZIS/N-HCS, via a combination of template etching and in situ growth strategies. Characterization results demonstrate that this hollow structure possesses a high specific surface area (48.41 m^2^/g) and a narrowed bandgap (2.41 eV), achieve broad-spectrum light absorption, thereby enabling the catalyst to generate a local hot spot temperature of 136 °C under AM1.5G conditions. The optimized ZIS/N-HCS-0.30 sample exhibited a significantly enhanced photocurrent response (8.26 μA cm^−2^) and improved charge separation efficiency. When evaluated at a set solution temperature of 20 °C, the material exhibited a photocatalytic hydrogen evolution rate of 17.03 mmol g^−1^·h^−1^, which is 7.06 times higher than that of pure ZIS. Furthermore, it demonstrated excellent cycling stability. This work elucidates the synergistic role of the hollow photothermal structure in enhancing solar energy utilization and catalytic reaction kinetics, providing a new strategy for designing efficient solar-driven hydrogen production systems.

## 1. Introduction

Rapid industrial development has driven societal progress but concurrently intensified critical challenges, including resource scarcity, energy crises, and environmental pollution [1]. Compounding these issues, global reserves of fossil fuels are diminishing. Their non-renewable nature promotes overexploitation and excessive consumption, thereby accelerating resource depletion and environmental degradation. In this context, solar energy emerges as a clean, sustainable, and naturally renewable resource. Its utilization represents an essential pathway for humanity to achieve sustainable development [2,3]. Photocatalytic hydrogen (H_2_) production by splitting water is the most promising way, as it can convert solar energy into chemical energy, circumventing the discontinuous and unstable utilization of solar energy [4].

The efficiency of a photocatalytic reaction is highly dependent on two key factors: the intrinsic properties of the photocatalyst and the external reaction conditions. Two-dimensional metal sulfides have attracted considerable research interest due to their large specific surface area, strong visible-light response, and short transport distance for photogenerated charge carriers [5,6]. The built-in electric field within its crystal exhibits excellent photogenerated charge separation efficiency. However, the low utilization rate of sunlight and the tendency to aggregate significantly limit its photocatalytic activity. To address these limitations, various strategies have been explored, including constructing heterojunctions [7], introducing defects [6], elemental doping [8], and morphology control [9,10]. For instance, Su et al. [11] constructed a ZnIn_2_S_4_-encapsulated TiO_2_ S-scheme core–shell heterojunction photocatalyst via a one-pot solvothermal strategy. This work demonstrates the potential of sophisticated heterojunction design to enhance charge separation efficiency. However, the inherently solid nature of their core–shell architecture entails long carrier transport paths, which can still lead to considerable bulk recombination. More critically, the system primarily relies on UV-vis light excitation and fails to efficiently utilize near-infrared (NIR) light, which accounts for approximately 50% of solar energy. This fundamentally constrains the upper limit of its full-spectrum solar conversion efficiency. In another study, Xiong et al. [12] pioneered a core–shell MoO_3−x_ @ZnIn_2_S_4_ composite, which achieved remarkable average CO and CH_4_ production rates of 4.65 and 28.3 mmol·g^−1^·h^−1^, respectively, under full-spectrum irradiation. This work represents a significant advancement in photothermal catalysis, confirming the feasibility of leveraging the photothermal effect to enhance catalytic performance. Nevertheless, a potential spatial decoupling between the “photothermal” and “catalytic” functions may exist, preventing the optimal utilization of thermal energy at the precise catalytic active sites.

Currently, most research indicates that hollow core–shell structures can shorten the transport distance of photogenerated carriers through geometric structure transformation and microenvironment regulation, effectively enhancing the separation of photogenerated carriers [13,14]. Moreover, the light-harvesting capability of hollow-structured catalysts is highly dependent on cavity curvature design and the management of scattered light absorption within the cavity, which critically determines the capture of high-energy photons at the exposed shell [15,16]. However, for most chalcogenide semiconductor photocatalysts, photocatalytic reactions typically utilize ultraviolet (UV) and visible (Vis) light. Their weak absorption in the visible and NIR regions—the latter contributing thermal effects—significantly limits the effective utilization of solar energy [17,18]. Recently, photothermal conversion has emerged as a feasible strategy that utilizes solar photons to generate heat, particularly in the near-infrared band. This approach can improve photocatalytic activity under relatively mild conditions, offering a novel pathway to address the aforementioned challenges. Consequently, the search for photothermal materials capable of efficiently and instantaneously converting solar energy into thermal energy—particularly those with absorption capabilities in both the visible and near-infrared regions—has become crucial, such as plasmonic metals [16,19], narrow-bandgap semiconductors [20], and carbon-based materials [21], and is of great importance. For example, Wang et al. [22] constructed a Cu_2−x_S@ZnIn_2_S_4_ S-scheme heterojunction using hollow Cu_2−x_S nanocages, which achieved an orders-of-magnitude enhancement in photothermal hydrogen evolution performance. This work convincingly demonstrates the feasibility of employing a hollow architecture for the photothermal component (Cu_2−x_S) to augment light absorption. In a separate study, Li et al. [23] designed a hollow hierarchical NiCo_2_O_4_@ZnIn_2_S_4_ structure, which explicitly introduced the concept of a local photothermal effect (LPE) and observed a substantial promotion of the hydrogen evolution rate. Collectively, these studies indicate that hollow structures not only enhance light scattering and absorption but also, by incorporating materials with superior photothermal conversion capability into the core region, can significantly strengthen the adsorption and activation of reactant molecules through tailored core composition and structure [24,25].

Nevertheless, NiCo_2_O_4_, the photothermal core of the material, is a metal oxide by nature. Its photothermal conversion efficiency generally relies on d-band electronic transitions. Compared with specialized carbon-based photothermal materials, its absorption capacity across a broad spectral range (especially in the NIR region) and the upper limit of photothermal conversion may be constrained. Therefore, among various core materials available for core–shell structures, carbon-based materials serve as ideal candidates due to their exceptional photothermal conversion efficiency and intrinsic electrical conductivity. Mi et al. [26] employed a carbon-doping strategy that not only introduced oxygen vacancies into the band gap of In_2_O_3_ but also enhanced its NIR light response. Cheng et al. [27] achieved in situ growth of Co_0.85_Se nanoparticles on sulfur vacancy-rich Mn_x_Cd_1−x_S nanorods. When the reaction temperature increased from 5 °C to 25 °C, the hydrogen evolution rate was enhanced by 1.7 times. Xia et al. [28] constructed a CdS/graphene nanoribbon hybrid system for thermal-assisted photocatalysis. The temperature rise induced by the photothermal effect in graphene promotes the migration of photogenerated charge carriers, thereby improving the photocatalytic activity. These results demonstrate that coupling carbon-based materials with semiconductor photocatalysts to construct NIR-driven photothermal-assisted systems represents an effective approach to broaden NIR light absorption and improve solar energy utilization. To enhance the optical absorption of carbon materials in the visible and NIR regions, an effective strategy involves expanding the sp^2^-conjugated domain within the carbon core of carbon quantum dots or introducing heteroatoms such as nitrogen (N) and sulfur (S) [29,30]. For instance, N-doped TiO_2_ can absorb the red portion of the spectrum, while C-doping improves the electrical conductivity of photocatalysts, facilitating the transport and separation of photogenerated charges [31]. This enhancement primarily originates from the significant differences in electronegativity and atomic radius between nitrogen (3.04) and carbon (2.55). Doping induces pronounced charge polarization on adjacent carbon atoms, which significantly reduces the bandgap [32].

In summary, although significant progress has been achieved in enhancing photocatalytic hydrogen production performance through strategies such as heterojunction construction on ZIS or photothermal unit introduction (Table S1), these systems still have obvious limitations. Therefore, in this work, to address the aforementioned challenges of limited light absorption and rapid charge recombination, this work strategically constructed a composite photocatalyst by integrating ZnIn_2_S_4_ (ZIS) with N-doped hollow carbon spheres (N-HCS). The unique hollow structure of N-HCS will not only enhance light harvesting through multiple internal reflections but also, coupled with its nitrogen-doped nature, serve as an efficient photothermal converter and electron acceptor. By deliberately leveraging the photothermal effect to overcome the inherent bottlenecks of traditional photocatalysis. Moreover, the mesoporous shell of the N-HCS allows free diffusion of reactants and products between the internal cavity and the external environment, thereby fully utilizing the high chemical reactivity within the nanoreactor and resulting in highly efficient photocatalytic hydrogen evolution.

## 2. Results and Discussion

**Scheme 1:**
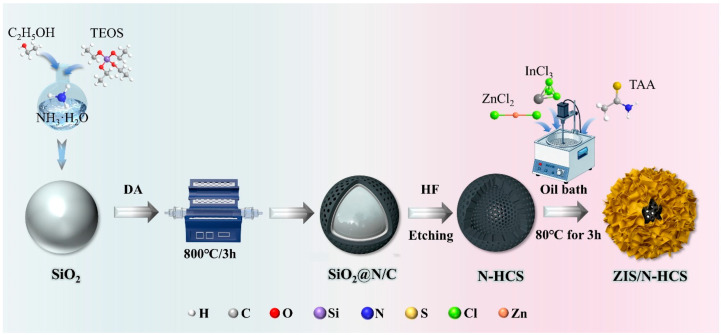
Experimental flowchart for the design of the ZIS/N-HCS.

The synthesis procedure of the ZIS/N-HCS composite is illustrated in Scheme 1. The ZIS/N-HCS composite was prepared via a one-step hydrothermal method at elevated temperature. Initially, the photothermal layer was grown on a sacrificial template, which was subsequently removed by etching. The photocatalytic ZIS layer was then deposited in a single hydrothermal step using a stainless-steel autoclave lined with Teflon.

**Figure 1:**
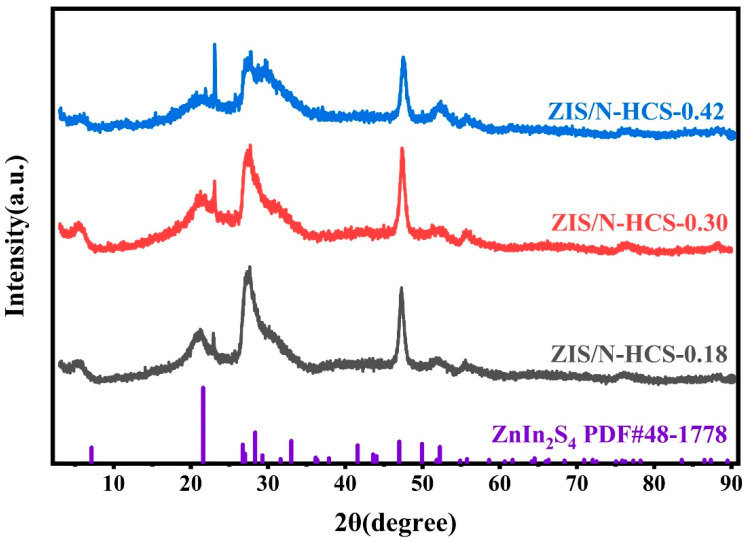
XRD patterns of pure ZIS and ZIS/N-HCS composites with different mass ratios.

Figure 1 presents the XRD patterns of ZIS/N-HCS composite catalysts with different mass ratios. All composite materials exhibit sharp and well-defined diffraction peaks, which correspond closely to the standard PDF#48-1778 card. This indicates that ZIS maintains a well-preserved crystalline structure after its growth on N-HCS, without a significant loss of crystallinity due to the composite formation. The characteristic diffraction peaks corresponding to the (006), (102), and (110) crystal planes of ZnIn_2_S_4_ are located at 21.8°, 27.6°, and 48.4°, respectively. It is evident that the intensity of the characteristic ZIS diffraction peaks gradually decreases with increasing N-HCS content. No distinct diffraction peaks attributable to N-HCS are detected in the composites, which is ascribed to its amorphous nature and low degree of crystallinity [33]. These XRD results confirm the successful synthesis of a series of ZIS/N-HCS composite photocatalysts with varying mass ratios.

**Figure 2:**
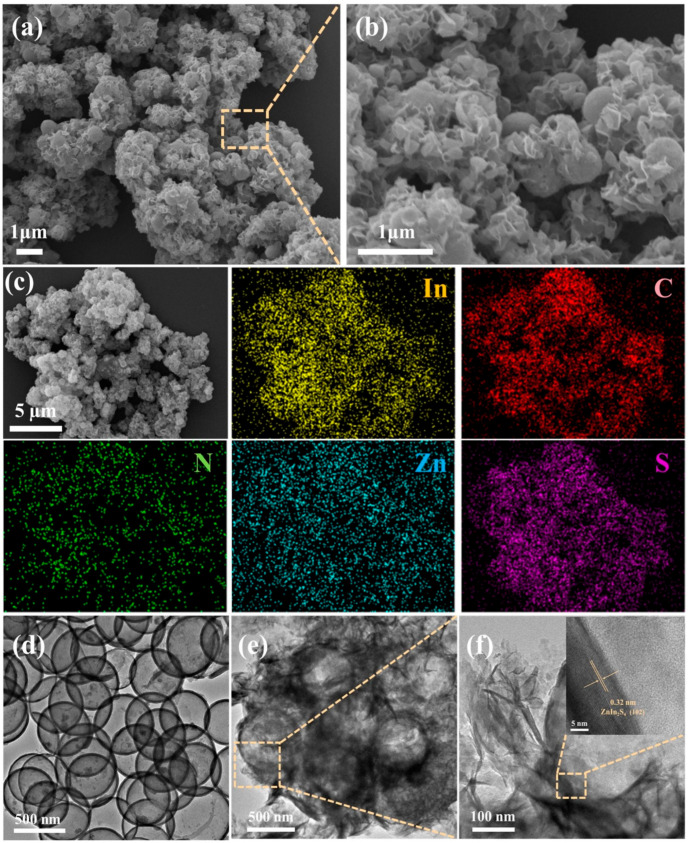
SEM (**a**,**b**) images of ZIS/N-HCS-0.30. HAADF-STEM image and EDS analysis of ZIS/N-HCS-0.30 (**c**). TEM image of N-HCS (**d**). TEM and HRTEM images of ZIS/N-HCS-0.30 (**e**,**f**).

To gain deeper insight into the microstructure of the synthesized catalysts, SEM was employed to characterize the morphology of the ZIS/N-HCS-based composites. As shown in Figure 2a, ZnIn_2_S_4_ is uniformly coated on the surface of the N-HCS spheres. Energy-dispersive X-ray spectroscopy (EDS) was used to systematically analyze the elemental composition and spatial distribution of the composite photocatalyst (Figure 2c). The EDS analysis of the composite reveals a homogeneous distribution of Zn, In, S, N, and C elements, with no extraneous elements detected. Furthermore, the hollow carbon spheres were confirmed to have a C to N atomic ratio of 3:1 (Figure S1). To further confirm the formation of the hollow structure and verify the growth of ZIS on the N-HCS surface, TEM was utilized to systematically characterize the microstructure of the ZIS/N-HCS-0.30 composite. High-resolution TEM (HRTEM) imaging clearly revealed the surface structural features and component distribution of the composite. Figure S2a,b demonstrates the successful preparation of the template and SiO_2_@N/C. Figure 2d clearly shows that after HF etching, uniform N-HCS with a diameter of approximately 500 nm was obtained, and the wall thickness was about 20 nm. This wall thickness ensured the effective removal of the SiO_2_ core while preserving its spherical morphology, thus providing ample space for the subsequent growth of ZIS with a diameter of approximately 700 nm. Subsequently, the HRTEM image in Figure 2f confirms the presence of a close heterojunction interface, where the measured lattice fringe spacing of 0.32 nm corresponds to the (102) crystal plane of ZIS. These findings are consistent with the XRD results, collectively confirming the successful fabrication of the composite catalyst.

**Figure 3:**
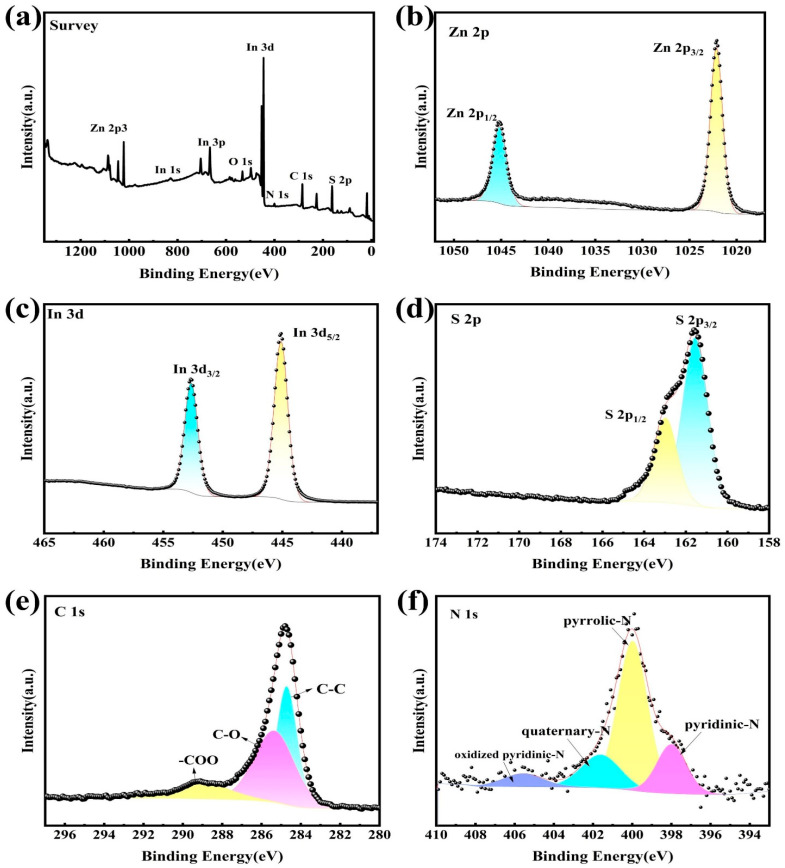
XPS survey spectra of ZIS/N-HCS-0.30 (**a**), High-resolution Zn 2p (**b**), In 3d (**c**), S 2p (**d**), C 1s (**e**), and N 1s (**f**) XPS spectra.

To gain deeper insight into the surface chemical properties of the ZIS/N-HCS-0.30 composite catalyst, XPS analysis was conducted to determine the elemental composition and chemical states of the constituent elements. The high-resolution XPS spectra of Zn 2p, In 3d, S 2p, C 1s, and N 1s for the composite catalyst are presented in Figure 3b–f. As shown in Figure 3b, the Zn 2p spectrum exhibits two distinct peaks at binding energies of 1045.28 eV and 1022.22 eV, corresponding to Zn 2p_1/2_ and Zn 2p_3/2_, respectively. This confirms the presence of Zn^2+^ in the catalyst. The high-resolution spectrum of In 3d (Figure 3c) shows two characteristic peaks at 445.15 eV (In 3d_5/2_) and 452.68 eV (In 3d_3/2_), which are consistent with the chemical state of In^3+^. Furthermore, the S 2p spectrum (Figure 3d) displays spin–orbit doublets at 161.46 eV (S 2p_3/2_) and 162.73 eV (S 2p_1/2_), further verifying the presence of S^2−^ in the catalyst. The C 1s spectrum (Figure 3e) can be deconvoluted into three components: a peak at 289.36 eV assigned to -COO groups, a peak at 285.23 eV corresponding to C-O bonds, and the main peak at 284.09 eV attributed to C-C bonds. The N 1s spectrum (Figure 3f) was fitted into four components: pyridinic N at 397.8 eV, pyrrolic N at 399.89 eV, quaternary N at 401.67 eV, and pyridinic N-oxide at 405.66 eV. The XPS results further confirm the successful preparation of the composite catalyst and lay the foundation for studying the photocatalytic mechanism.

**Figure 4:**
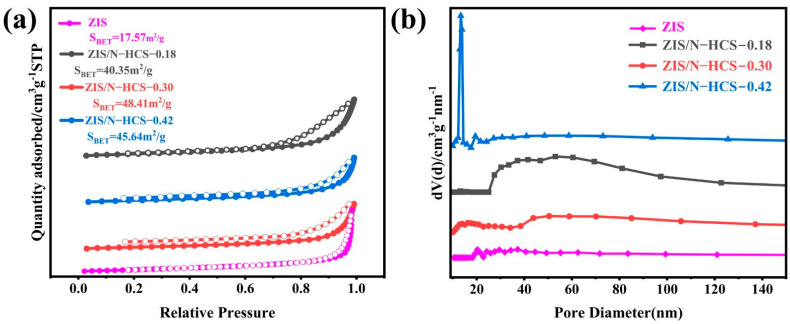
N_2_ adsorption–desorption isotherms (**a**). Barrett−Joyner−Halenda (BJH) pore size distributions (**b**).

The N_2_ adsorption–desorption isotherms (Figure 4a) and Table 1 reveal that the ZIS/N-HCS-0.30 composite possesses a significantly larger specific surface area (48.41 m^2^/g) compared to pure ZIS (17.57 m^2^/g), which is attributed to the intrinsically porous and hollow architecture of the N-HCS support. This enhancement effect is attributed to the inherent high specific surface area and porous structure of N-HCS, which are crucial for providing abundant active sites for the hydrogen evolution reaction [27,29]. However, an excessive amount of N-HCS may lead to non-uniform dispersion and agglomeration of ZIS, consequently reducing the effective surface area (45.64 m^2^/g for ZIS/N-HCS-0.42) [22]. The BJH pore size distribution curves (Figure 4b) indicate that the pores in the ZIS/N-HCS composites are primarily distributed in the range of 40–90 nm. However, the numerical average pore diameter calculated from the desorption branch differs due to the presence of larger macropores beyond this range, which significantly influence the average value, indicating the presence of abundant mesoporous structures across the series. In summary, the combination of a large specific surface area and well-defined mesoporosity in these samples facilitates the exposure of more active sites, which is beneficial for enhancing the overall catalytic activity.

**Figure 5:**
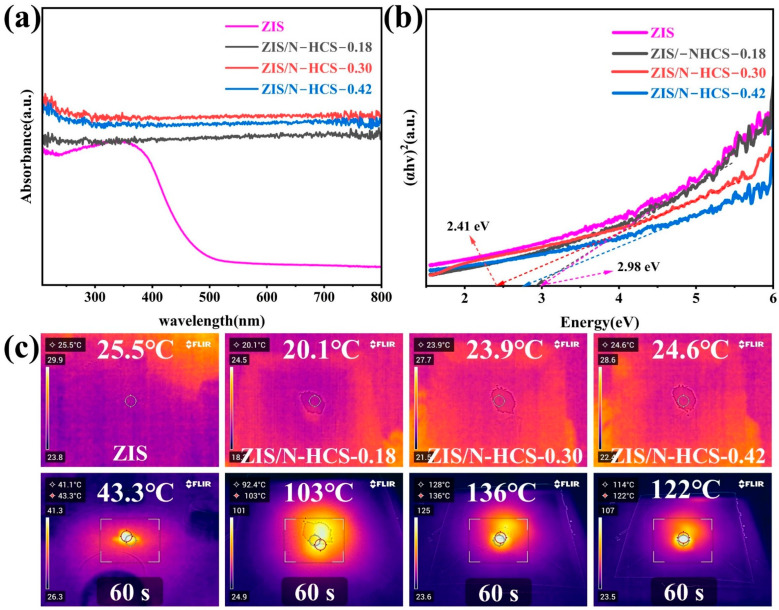
UV-Vis DRS absorption spectrum (**a**) and absorption spectrum obtained by Kubelka-Munk transformation function (**b**). Local hotspot IR thermal images of ZIS, ZIS/N-HCS-0.18, ZIS/N-HCS-0.30, ZIS/N-HCS-0.42 powders before and after irradiation for 60 s (**c**).

Light absorption capability is a critical factor influencing catalyst performance. The UV-Vis diffuse reflectance spectra of the samples were obtained over the 200–800 nm wavelength range, as shown in Figure 5a. The pure ZIS sample exhibits an absorption edge at around 500 nm, indicating its response is confined primarily to the visible light region. In contrast, the ZIS/N-HCS-based materials appear black due to their nearly complete absorption across the entire 200–800 nm spectrum. This broad-spectrum response promotes greater charge carrier generation and enhances photothermal conversion. Furthermore, the construction of the hollow structure enhances light absorption intensity across the entire spectrum. The internal cavity facilitates multiple internal reflections and scattering of incident light, mitigating light relaxation losses and thereby improving the overall utilization efficiency of the full spectrum. To further verify the role of ZIS/N-HCS in photothermal enhancement, infrared thermal imaging analysis was performed on the prepared samples under full-spectrum irradiation. As shown in Figure 5c and Figure S3, under AM 1.5G illumination, the temperature of each sample gradually increased over a 60 s exposure period. The surface temperature of pure ZIS rose only marginally, reaching 43.3 °C. Notably, the surface temperature of ZIS/N-HCS-0.30 reached 136 °C. This significant rise is attributed to the low thermal conductivity of the hollow cavities, which minimizes heat dissipation to the surroundings. Consequently, the heat generated from photothermal conversion accumulates near the catalyst surface and active sites, creating a localized high-temperature zone that is conducive to accelerating reaction kinetics. When the N-HCS content is excessively high, although light absorption may be further enhanced, the dispersion of ZIS deteriorates, leading to the coverage of active sites and reduced separation efficiency of photogenerated charge carriers. Additionally, an overabundance of carbon material can disrupt efficient heat conduction pathways, preventing heat from being effectively concentrated at the catalytically active sites and thus limiting the overall temperature increase. Conversely, when the N-HCS content is too low, the number of hollow structures is correspondingly scarce, leaving most areas as solid ZIS aggregates. Heat readily dissipates through the solid material into the surrounding solution, preventing effective heat accumulation. Therefore, the lack of sufficient thermally insulating chambers to trap and concentrate heat results in a negligible overall temperature rise. In summary, ZIS/N-HCS-0.30 achieves the highest temperature by maximizing both the absorption of light for heat generation and the utilization of its hollow structure to effectively concentrate this thermal energy.

**Figure 6:**
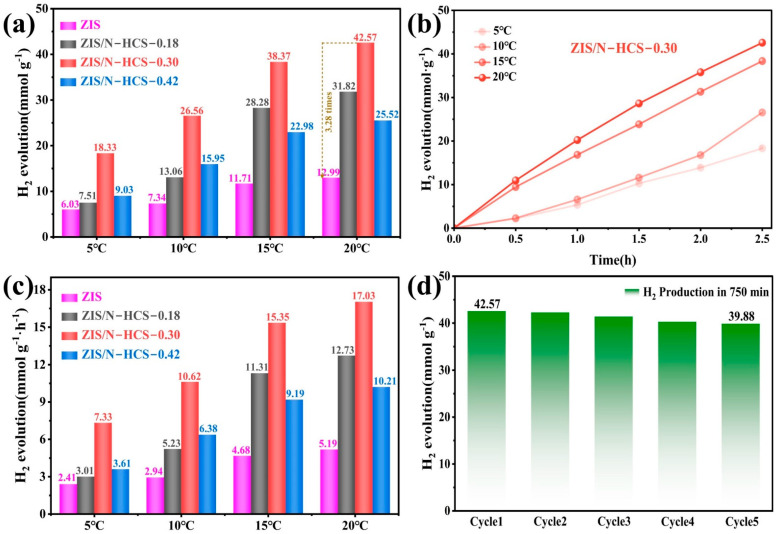
Hydrogen evolution quantities of all samples at different solution set temperatures (**a**), time evolution of the photothermal catalytic performance of ZIS/N-HCS-0.30 at various reaction temperatures (**b**), H_2_ production rates of ZIS, ZIS/N-HCS-0.18, ZIS/N-HCS-0.30 and ZIS/N-HCS-0.42 at different reaction temperatures (**c**), and cycling stability test of ZIS/N-HCS-0.30 within 750 min (**d**).

In the photothermal catalytic process, the enhanced photothermal effect plays a critical regulatory role: it significantly intensifies the kinetic activity of internal particles, accelerates the kinetics of charge carrier generation, and optimizes electron transfer efficiency within the built-in electric field. These synergistic modulations collectively contribute to a substantial improvement in performance. To elucidate the influence of the photothermal effect on hydrogen production, temperature-dependent photocatalytic experiments were conducted on ZIS-based photocatalysts. As shown in Figure 6a,b, the hydrogen evolution performance of all samples exhibited a positive correlation with the reaction temperature. For the ZIS/N-HCS-0.30 material, when the set solution temperature was raised to 20 °C, the hydrogen evolution rate increased to 17.03 mmol g^−1^·h^−1^ within 2.5 h. Its performance was not only 7.06 times higher than that of pure ZIS, but also performed exceptionally well compared to other advanced ZnIn_2_S_4_-based composite materials reported recently (Table S2). In contrast, the hydrogen production of pure ZIS increased by only 2.15 times under the same temperature change. Regarding the introduction of varying amounts of N-HCS, the optimal thermal conduction pathways and active sites in ZIS/N-HCS-0.30 resulted in a superior hydrogen evolution rate compared to ZIS/N-HCS-0.18 and ZIS/N-HCS-0.42 (Figure 6c and Figure S4). During the 750 min cycling test, ZIS/N-HCS-0.30 exhibited no significant performance degradation, demonstrating the excellent stability of the hollow structure (Figure 6d). These temperature-dependent photocatalytic experiments confirm that the incorporation of N-HCS significantly enhances photocatalytic performance, attributable to its broad-spectrum absorption, rapid charge separation, and remarkable photothermal conversion capability.

**Figure 7:**
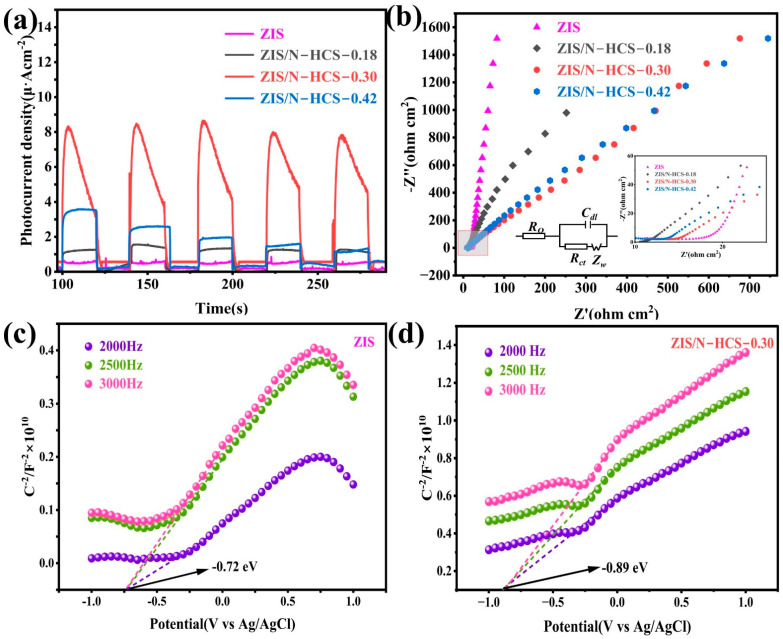
Transient photocurrent responses (**a**), EIS profiles (**b**) of ZIS, ZIS/N-HCS-0.18, ZIS/N-HCS-0.30 and ZIS/N-HCS-0.42. Mott-Schottky (M-S) diagrams of ZIS (**c**) and ZIS/N-HCS-0.30 (**d**).

To further investigate the charge separation efficiency and transfer kinetics of the catalysts, photoelectrochemical measurements were conducted under irradiation from a 300 W xenon lamp. The tests were systematically performed on ZIS and the composite samples in a 0.5 mol/L Na_2_SO_4_ aqueous solution at a bias voltage of 0.5 V vs. Ag/AgCl. As shown in Figure 7a, the photocurrent response of all samples was nearly negligible in the dark. Under 300 W xenon lamp irradiation, ZIS/N-HCS-0.30 exhibited the highest transient photocurrent density of 8.26 μA cm^−2^, which is 17.2 times greater than that of pure ZIS. This result indicates that ZIS/N-HCS-0.30 can provide more photogenerated electrons for the reduction reaction and highlights the crucial role of N-HCS in extracting and transmitting photogenerated electrons. Furthermore, electrochemical impedance spectroscopy (EIS) was employed to characterize the charge transfer efficiency. As depicted in Figure 7b, ZIS/N-HCS-0.30 displays the smallest Nyquist plot radius, indicating the lowest charge transfer resistance. The inset shows the corresponding equivalent circuit model, where R_Ω_ and R_ct_ represent the electrolyte resistance and charge transfer resistance, respectively, C_dl_ is the double-layer capacitance, and Z_w_ is the Warburg impedance. To gain deeper insight into the carrier separation efficiency of the ZIS/N-HCS composites, photoluminescence (PL) spectroscopy was performed. As shown in Figure S5, ZIS/N-HCS-0.30 exhibits significant PL quenching, indicating effective suppression of electron-hole recombination and a prolonged charge carrier lifetime. These findings collectively demonstrate that the introduction of N-HCS promotes the migration kinetics of photoinduced charge carriers. The ZIS/N-HCS-0.30 composite effectively accelerates the separation and transport of photogenerated charges, thereby enhancing the performance of photothermal catalytic hydrogen evolution.

The bandgap energy of ZIS/N-HCS-0.30 was precisely determined by combining the Kubelka-Munk theory [32] with Tauc plot analysis. As shown in Figure 5b, the band gap of pure ZIS is 2.98 eV, while the apparent optical band gap of the ZIS/N-HCS-0.30 composite material decreases to 2.41 eV. This observed reduction is primarily attributed to the strong broadband absorption of the N-HCS component and enhanced light scattering within the hollow structure, which collectively improve the light-harvesting efficiency of the composite material and benefit the photothermal catalytic hydrogen evolution reaction. To elucidate the photocatalytic mechanism in detail, the valence band (E_VB_) and conduction band (E_CB_) positions of ZIS/N-HCS-0.30 were determined. Mott-Schottky analysis was employed to investigate the differences in the band structures of the prepared samples (Figure 7c,d), confirming the electronic modulation effect and the charge carrier migration behavior within the composite. The flat-band potentials (vs. Ag/AgCl) of ZIS and ZIS/N-HCS-0.30 were measured at different frequencies, yielding values of −0.72 eV and −0.89 eV, respectively. These values were converted to −0.52 eV and −0.69 eV versus the normal hydrogen electrode (NHE). The positive slopes of the linear plots confirm the n-type semiconductor behavior of ZIS/N-HCS-0.30. Since the flat-band potential for n-type semiconductors is typically approximately 0.1 eV higher than the E_CB_ potential [33], the E_CB_ of ZIS/N-HCS-0.30 was calculated to be −0.79 eV (vs. NHE). Consequently, based on its bandgap energy (Figure 5b), the E_VB_ of ZIS/N-HCS-0.30 was determined to be 1.62 eV (vs. NHE). These results demonstrate that the superior light absorption capability of ZIS/N-HCS-0.30 is attributed to the introduction of N-HCS.

**Figure 8:**
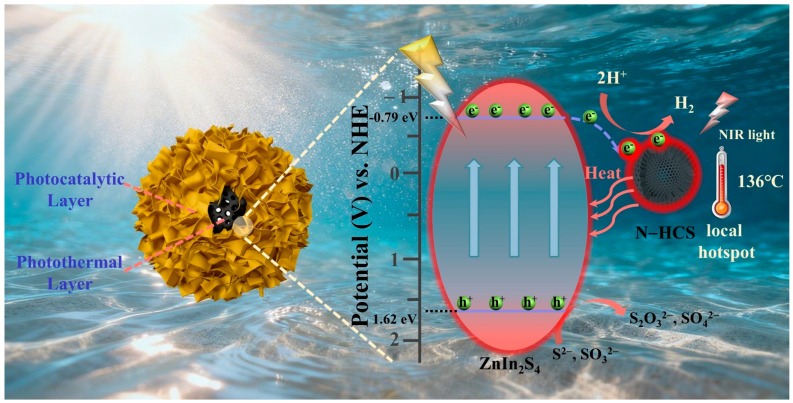
Diagram of a possible photothermal catalytic mechanism of ZIS/N-HCS composites.

Based on the aforementioned results, Figure 8 illustrates the proposed band structure and photothermal catalytic mechanism of the ZIS/N-HCS-0.30 composite. Under full-spectrum irradiation, the photocatalytic component (ZIS) absorbs photons and generates electron-hole pairs, which subsequently drive the water splitting reaction. Simultaneously, the photothermal component (N-HCS), owing to its broad-spectrum absorption capability, converts photon energy into thermal energy via non-radiative relaxation. The hollow cavity structure of N-HCS acts as a thermal insulator, confining the generated heat and creating a localized high-temperature region within the material. This localized heating serves a dual purpose: firstly, it thermally activates and significantly accelerates the surface reaction kinetics. Secondly, it thermally excites electrons in the adjacent ZIS semiconductor, enhancing intrinsic carrier generation and markedly increasing the migration rate of the photogenerated electrons. Furthermore, the intimate heterojunction interface formed between N-HCS and ZIS facilitates the spatial separation of photogenerated charge carriers. Specifically, electrons are efficiently transferred from the CB of ZIS to N-HCS, thereby substantially suppressing the recombination of electron-hole pairs. Concurrently, the photogenerated holes are efficiently consumed by reacting with sacrificial agents (S^2−^/SO_3_^2−^) present in the aqueous solution. The synergistic interplay of these mechanisms collectively enables the significant enhancement in photothermal-assisted photocatalytic hydrogen evolution performance observed for this material.

## 3. Experimental

### 3.1. Reagents and Instruments

Zinc chloride (ZnCl_2_), indium chloride tetrahydrate (InCl_3_·4H_2_O), thioacetamide (TAA), absolute ethanol, and tetraethyl orthosilicate (TEOS) were all purchased from Macklin Biochemical Co., Ltd. (Shanghai, China). Ammonia solution was obtained from Luoyang Haohua Chemical Reagent Co., Ltd. (Henan, China). Tris-HCl buffer was sourced from Xiamen Aimeimanni Biotechnology Co., Ltd. (Fujian, China). Dopamine hydrochloride (C_8_H_11_NO_2_·HCl, 98%) was supplied by Shandong Keyuan Biochemical Co., Ltd. (Shandong, China). Hydrofluoric acid (HF) and hydrochloric acid (HCl) were acquired from Shanghai Aladdin Biochemical Technology Co., Ltd. (Shanghai, China). All aqueous solutions were prepared using deionized water produced in-house.

### 3.2. Synthesis of SiO_2_@PDA

The Stober method was used to synthesize SiO_2_ balls [34]. N-HCS were synthesized using SiO_2_ nanospheres (450 nm) as templates. Typically, 5.6 mL of TEOS was added to a mixture containing 60 mL of ethanol, 10 mL of deionized water, and 5.5 mL of ammonia solution. After stirring for 1 h, the product was collected by centrifugation and dried at 60 °C to obtain the SiO_2_ template. Subsequently, 100 mg of the as-synthesized SiO_2_ template was dispersed in 80 mL of Tris-HCl buffer via ultrasonication for 30 min. Then, 80 mg of dopamine hydrochloride was introduced, and the reaction was allowed to proceed under stirring for 12 h in the absence of light. Finally, the resulting product was collected by centrifugation, dried at 60 °C for 5 h, and denoted as SiO_2_@PDA.

### 3.3. Synthesis of ZnIn_2_S_4_ Grown on N-Doped Hollow Carbon Spheres (ZIS/N-HCS)

First, 100 mg of SiO_2_@PDA was placed in a ceramic boat and calcined at 800 °C for 3 h under an Ar atmosphere to obtain SiO_2_@N/C. The resulting carbonized product was then stirred in a 10% HF solution for 2 h to etch away the SiO_2_ template, yielding N-HCS. Next, 30 mg of the as-prepared N-HCS was dispersed in 100 mL of acidified deionized water. To this dispersion, 272 mg of ZnCl_2_, 442 mg of InCl_3_·4H_2_O, and 300 mg of thioacetamide (TAA) were sequentially added. The mixture was then stirred and reacted at 80 °C in an oil bath for 3 h. The final product was collected, washed with ethanol and deionized water, and dried at 60 °C, resulting in the composite designated as ZIS/N-HCS-0.30. To optimize the material’s properties, control samples were synthesized by varying the mass of N-HCS to 18 mg and 42 mg. These samples were labeled as ZIS/N-HCS-0.18 and ZIS/N-HCS-0.42, respectively.

### 3.4. Experiments on Photothermal-Assisted Photocatalytic Hydrogen Production

To assess the hydrogen evolution performance, photothermal catalytic H_2_ production experiments were carried out in an online closed-loop gas analysis system (Meiruichen, Beijing, China, model MC-SCO2IIAG) coupled with a 300 W Xe lamp and an AM 1.5G filter. The reaction was performed under vacuum in a sealed quartz reactor containing 15 mg of catalyst dispersed in 50 mL of an aqueous solution with 0.2 M Na_2_SO_3_ and 0.2 M Na_2_SO_4_ as sacrificial agents. The irradiation intensity was maintained at 150 mW/cm^2^. The amount of evolved hydrogen was monitored at 30 min intervals via gas chromatography (GC). A five-point external standard calibration curve was constructed to correlate GC peak areas with H_2_ quantities, allowing for accurate yield determination. To examine the temperature dependence of the catalytic activity, experiments were conducted at controlled water temperatures (5 °C, 10 °C, 15 °C, and 20 °C) using a recirculating chiller. For stability evaluation, the catalyst was recovered after each reaction cycle by centrifugation, washed repeatedly with deionized water and ethanol to eliminate surface residues, dried, and then reused in subsequent tests with freshly prepared sacrificial solutions.

## 4. Conclusions

In summary, this study successfully achieved synergy between photothermal effects and photocatalytic processes by constructing a composite catalytic system with ZIS supported on N-HCS. This design ingeniously integrates the geometric advantages of the hollow structure with N-HCS, which serves a dual function as both a photothermal conversion center and an electron acceptor. The optimized ZIS/N-HCS-0.30 sample demonstrated exceptional performance, evidenced by a remarkable photocatalytic hydrogen evolution rate of 17.03 mmol g^−1^·h^−1^, which is 7.06 times higher than that of pure ZIS. This superior activity is directly supported by the following key findings: (1) The unique hollow structure endowed the composite with a high specific surface area of 48.41 m^2^/g, providing abundant active sites; (2) The incorporation of N-HCS induced apparent optical band gap narrowing to 2.41 eV, extending absorption well into the visible and near-infrared, which supports simultaneous photothermal heating and photocatalysis and generating a local hot spot temperature of 136 °C under illumination, which significantly accelerated reaction kinetics; (3) The intimate heterojunction interface facilitated efficient charge separation, as confirmed by a photocurrent density 17.2 times greater than that of pure ZIS and the smallest charge transfer resistance in EIS analysis. This design transcends conventional component doping or structural modifications by actively utilizing the photothermal effect to overcome intrinsic limitations of photocatalysis, namely, low charge separation efficiency and poor utilization of the solar spectrum. Therefore, this work not only provides a high-performance catalyst but also establishes a new paradigm for designing efficient solar-driven hydrogen production systems through the strategic integration of photothermal and photocatalytic principles.

## Figures and Tables

**Table 1:** SSA, pore size, and pore volume of various catalysts.

Sample	BET Surface Area (m^2^/g)	Pore Volume (cm^3^/g)	Average Pore Diameter (nm)
ZIS	17.57	0.13	14.2
ZIS/N-HCS-0.18	40.35	0.19	94.4
ZIS/N-HCS-0.30	48.41	0.24	98.4
ZIS/N-HCS-0.42	45.64	0.19	83.4

## Data Availability

Data are contained within the article and Supplementary Materials.

## Supplementary Materials

The following supporting information can be downloaded at: https://www.mdpi.com/article/10.3390/molecules30224368/s1. Figure S1: EDS element content of ZIS/N-HCS-0.30; Figure S2: TEM images of: (a) SiO_2_, (b)SiO_2_@N/; Figure S3: Infrared thermal images of powder samples under 60 s of irradiation: (a) ZIS, (b) ZIS/N-HCS-0.18, (c) ZIS/N-HCS-0.30, and (d) ZIS/N-HCS-0.42; Figure S4: Evolution rate diagram of photothermal catalytic performance over time at different reaction temperatures; Figure S5: PL spectra of (a) ZIS, (b) ZIS/N-HCS-0.18, (c) ZIS/N-HCS-0.30, and (d) ZIS/N-HCS-0.42; Table S1: A Comparative Study of This Work with Contemporary Systems for Photothermal Hydrogen Production; Table S2: Comparison of the catalytic hydrogen evolution rates achieved in this work with those reported for other ZnIn_2_S_4_-based catalysts in the literature. Refs. [35,36,37,38,39,40,41,42,43,44,45,46] are cited in Supplementary Materials.
