# Supplementary material for: Coupling Photothermal Effect in N-Doped Hollow Carbon Spheres with ZnIn2S4 Boosts Solar Hydrogen Evolution
Source: Molecules. 2025 Nov 12;30(22):4368. doi: 10.3390/molecules30224368 (PMC12655824; doi:10.3390/molecules30224368)
Supplement: Supplementary file 1 [file molecules-30-04368-s001.zip › molecules-3953588-supplementary.pdf]

Supporting information

Coupling Photothermal Effect in N-Doped Hollow Carbon Spheres with $ZnIn_2S_4$ Boosts Solar Hydrogen Evolution

Shanhao He [1,3], Li Liu [2], Min Liu [1], Jinjun Tian [1], Yan Xue [1*], Keliang Wu [1]

1. School of Biology and chemical Engineering/Henan Key Laboratory of microbialfermentation, Nanyang Institute of Technology, Henan, Nanyang, 473000, PR China;
   18239373350@163.com (S.H.); tianjinjun2005@163.com (J.T.);
   17709960825@163.com (L.L.); liumin031112@163.com (M.L.);
   wukeliang@nyist.edu.cn (K.W.)
2. Bayingolin Vocational and Technical College, Korla, Bayingolin Mongol Autonomous Prefecture, Xinjiang, 841000, PR China.
3. School of Chemistry and Chemical Engineering/State Key Laboratory Incubation Base for Green Processing of Chemical Engineering, Shihezi, Xinjiang 832003, P. R. China.

* **Correspondence:** Xueyannew@126.com (Y.X.)

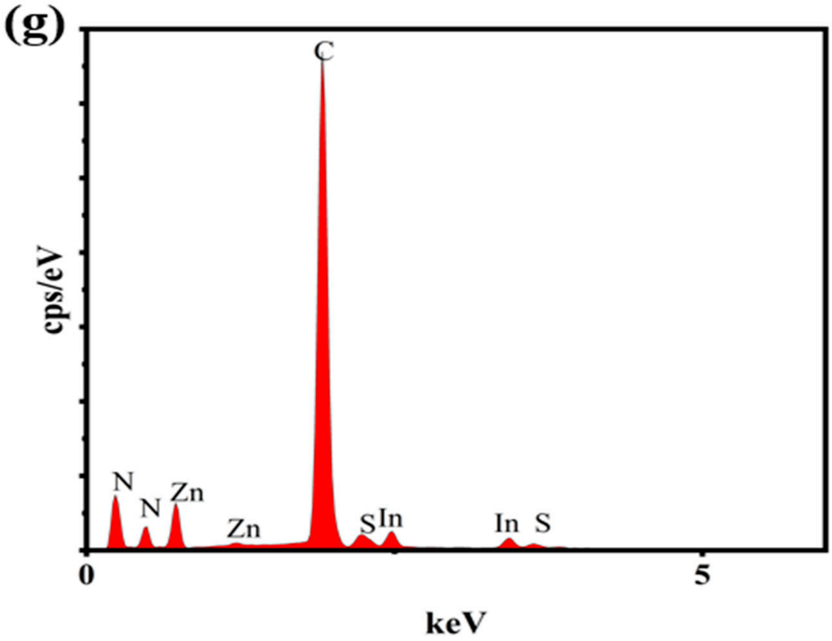

Supplementary Figure S1. EDS element content of ZIS/N-HCS-0.30.

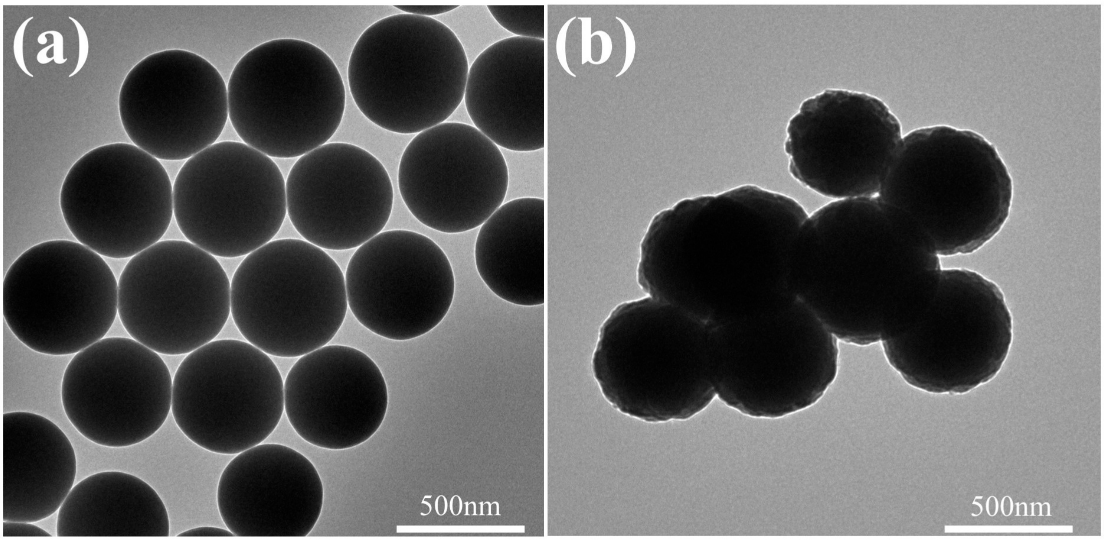

Supplementary Figure S2. TEM images of: (a) SiO_2, (b)SiO_2@N/C.

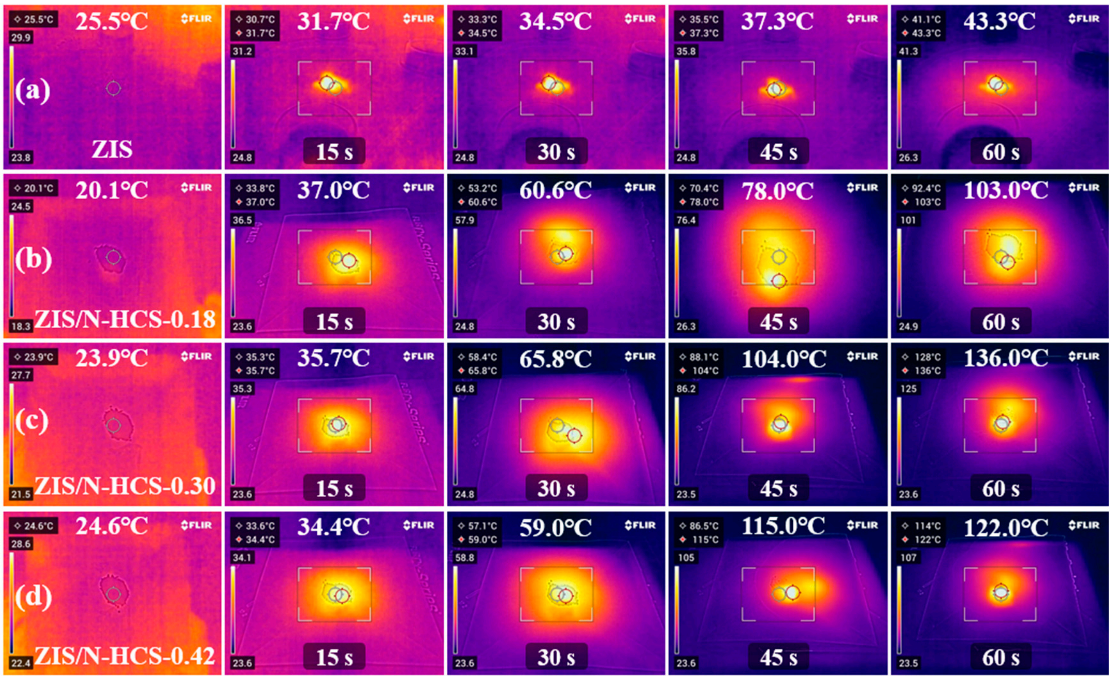

Supplementary Figure S3. Infrared thermal images of powder samples under 60 s of irradiation: (a) ZIS, (b) ZIS/N-HCS-0.18, (c) ZIS/N-HCS-0.30, and (d) ZIS/N-HCS-0.42.

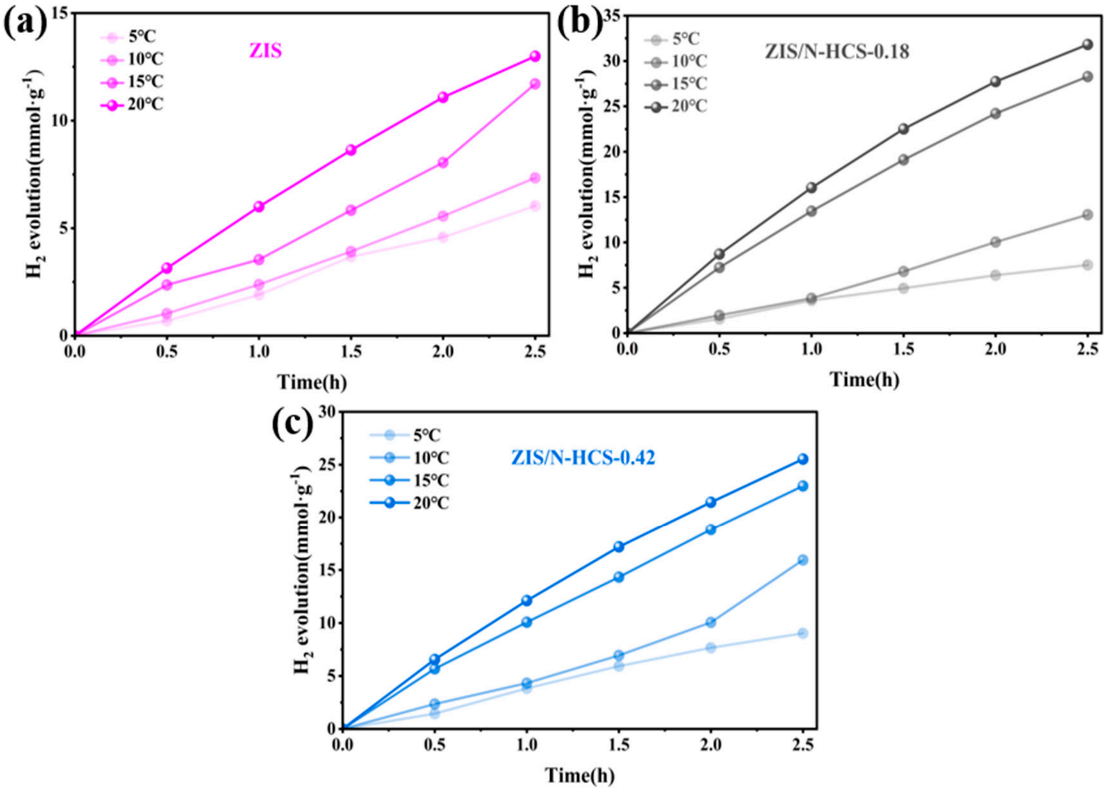

Supplementary Figure S4. Evolution rate diagram of photothermal catalytic performance over time at different reaction temperatures.

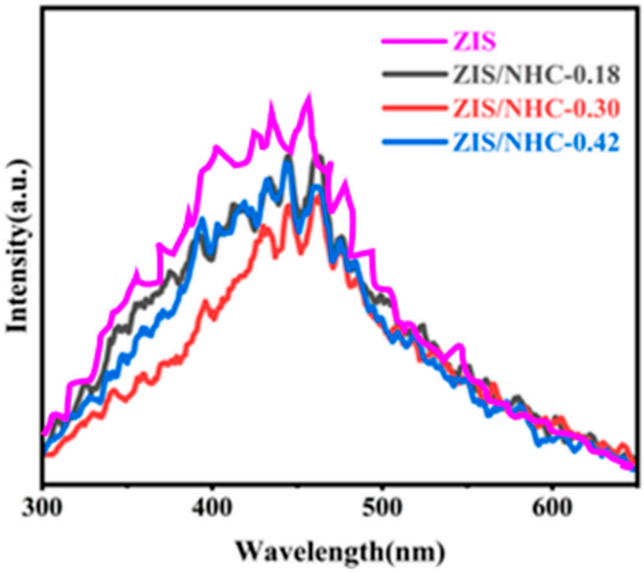

Supplementary Figure S5. PL spectra of (a) ZIS, (b) ZIS/N-HCS-0.18, (c) ZIS/N-HCS-0.30, and (d) ZIS/N-HCS-0.42.

Supplementary Table S1. A Comparative Study of This Work with Contemporary Systems for Photothermal Hydrogen Production.

Catalyst System	Core Mechanism	Advantages and Limitations
Su et al. [1]: TiO_2@ZIS	S-type heterojunction (core-shell)	It exhibits high charge separation efficiency; however, its solid structure cannot leverage the photothermal effect
Wang et al. [2]: $Cu_{2-x}S$@ZIS	S-shaped heterojunction + plasma photothermal	The material features a hollow plasmonic structure; however, the potential instability of the metal sulfide component remains a concern.
Li et al. [3]: $NiCo_2O_4$@ZIS	S-shaped heterojunction + local photothermal effect	It demonstrates excellent overall performance; however, its photothermal conversion capability is constrained by a fundamental limit.
**This Work:** ZIS/N-HCS	Hollow structure photothermal synergy + heterojunction charge separation	Strong upper limit of photothermal conversion, efficient charge separation, and excellent thermal localization work in synergy

Supplementary Table S2. Comparison of the catalytic hydrogen evolution rates achieved in this work with those reported for other $ZnIn_2S_4$-based catalysts in the literature.

**Photocatalyst**	**Simulated sunlight**	**Sacrificial agent**	**HER [mmol h^{-1} g^{-1}]**	**Ref.**
$CoP/ZnIn_2S_4$-4	AM1.5G	TEOA	4.24	[4]
Mo_2C-Pt@$ZnIn_2S_4$	AM1.5G	Lactic acid	8.04	[5]
$Ni_3C/ZnIn_2S_4$	AM1.5G	TEOA	7.68	[6]
Cu@C-N@$ZnIn_2S_4$	AM1.5G	TEOA	6.05	[7]

$W_{18}O_{49}$@$ZnIn_2S_4$	AM1.5G	TEOA	4.98	[8]
$ZnIn_2S_4$@Au@$Cd_{0.7}Zn_{0.3}S$	AM1.5G	Ethyl alcohol	4.57	[9]
Carbon@$ZnIn_2S_4$	AM1.5G	TEOA	2.97	[10]
Co_9S_8/$ZnIn_2S_4$	AM1.5G	TEOA	12.67	[11]
$ZnIn_2S_4$/MoO_{3-x}	AM1.5G	TEOA	11.08	[12]
CMT@TiO_2/$ZnIn_2S_4$	AM1.5G	TEOA	9.71	[13]
CDs/Ni_3P/$ZnIn_2S_4$	AM1.5G	EDTA	1.88	[14]
$FeSe_2$@$ZnIn_2S_4$	AM1.5G	EDTA	7.64	[15]
ZIS/N-HCS-0.30	AM1.5G	Na_2S/Na_2SO_3	17.03	**This Work**

Characterization of Products

1: Characterization Methods

All reported performance data represent the average of at least three independent experiments, with a deviation of less than 5% between replicates. The crystal structures of the as-prepared materials were characterized by X-ray diffraction (XRD) using a Rigaku MiniFlex600 diffractometer (Japan). The morphologies and microstructures were analyzed using a JSM-7900F scanning electron microscope (SEM) and a JEM-2100PLUS transmission electron microscope (TEM), both from JEOL Ltd., Japan. The specific surface area, pore structure parameters, and pore size distribution were determined by nitrogen physisorption measurements at 77 K, performed on a SSA-4300 analyzer (Beijing Builder Electronic Technology Co., Ltd., China). Electrochemical properties were measured with a CHI760E electrochemical workstation (Shanghai Chenhua Instrument Co., Ltd., China). The surface chemical composition and elemental states were investigated by X-ray photoelectron spectroscopy (XPS) on an Amicus ESCA 3200 spectrometer. All XPS spectra were calibrated by referencing the C 1s peak to a binding energy of 284.8 eV. UV-visible diffuse reflectance spectroscopy (UV-Vis DRS) was conducted on a Shimadzu UV-2700i spectrophotometer to evaluate the optical absorption properties. The surface temperature distribution and infrared radiation of the samples under illumination were monitored and visualized using a FLIR C3-X infrared thermal imaging camera.

2: Measurement of photothermal properties

The temperature variation of the synthesized samples was monitored under AM 1.5G illumination using an infrared thermal camera (FLIR C3-X). For solid surface temperature measurements, data were acquired every 15 s during light exposure.

3: Electrochemical performance test

Electrochemical measurements were conducted using a CHI760E electrochemical workstation with a standard three-electrode configuration, comprising a platinum plate counter electrode, a saturated Ag/AgCl reference electrode, and the as-prepared sample

as the working electrode. The measurements were performed in a 0.5 mol/L Na_2SO_4 aqueous solution under light irradiation with an intensity of 150 mW/cm^2, provided by a light source positioned 20 cm from the reaction cell. During the experiment, FTO (fluorine-doped tin oxide) conductive glass slides of appropriate size were carefully selected and subjected to ultrasonic cleaning with ethanol and deionized water to remove surface impurities. Subsequently, the slides were further treated with a surfactant via ultrasonication to enhance the cleaning effect. Finally, the glass slides were dried in an oven to ensure the cleanliness of the experimental materials and the accuracy of subsequent experiments. Sample preparation method: Catalysts were weighed out at 2 mg each and ultrasonically dispersed in a mixture of 0.45 mL of ethanol and 50 μL of 5 wt% Nafion solution. The entire suspension was then dropped onto a 1 cm^2 FTO conductive glass slide and allowed to evaporate before use.

References

[1] H. Su, Y. Gong, H. Lou, Y. Pang, D. Yang, D. Gao, X. Qiu, The open core-shell TiO_2@$ZnIn_2S_4$ step-scheme heterojunction to enhance mass transfer and light utilization for efficient photocatalytic performance. *J. Cleaner Prod.* **2023**, *419*, 138034.

[2] Y. Wang, M. Liu, C. Wu, J. Gao, M. Li, Z. Xing, Z. Li, W. Zhou, Hollow Nanoboxes $Cu_{2-x}S$@$ZnIn_2S_4$ Core-Shell S-Scheme Heterojunction with Broad-Spectrum Response and Enhanced Photothermal-Photocatalytic Performance. *Small.* **2022**, *18*, 2202544.

[3] W. Li, G. Zuo, S. Ma, L. Xi, C. Ouyang, M. He, Y. Wang, Q. Ji, S. Yang, W. Zhu, K. Zhang, H. He, Localized photothermal effect mediated hollow S-scheme $NiCo_2O_4$@$ZnIn_2S_4$ for enhanced photocatalytic hydrogen evolution. *Appl. Catal. B* **2025**, *365*, 124971.

[4] S. Li, J. Ye, Z. Fan, Y. Dai, Y. Xie, Y. Ling, M. Xu, Y. Wang, CoP co-catalyst modification $ZnIn_2S_4$ driving efficient H_2 evolution under visible light. *Sep. Purif. Technol.* **2025**, *358*, 130294.

[5] Y. Gao, F. Ren, Y. Lu, S. Zhang, P. Guo, J. Zhang, F. Dai, L. Chen, Y. Zhao, Mo_2C-supported ultralow-Pt-loading bifunctional cocatalyst for enhancing photocatalytic H_2 evolution on $ZnIn_2S_4$. *Sep. Purif. Technol.* **2025**, *377*, 133869.

[6] X. Liu, W. Gong, Z. Yan, Y. Wang, H. Guo, Y. Luo, Y. Li, J. Lin, Noble-metal-free Ni_3C cocatalysts decorated $ZnIn_2S_4$ nanosheet microspheres for improved photocatalytic H_2 evolution. *Int. J. Hydrogen Energy* **2025**, *150*, 150136.
[7] K. Geng, P. Shan, J. Shi, Y. Shen, S. Yan, M. Zhao, F. Guo, G. Wang, W. Shi, Broad spectral absorption cooperates with local plasma resonance for promoted photothermal-assisted photocatalytic hydrogen production. *Chem. Eng. J.* **2025**, *507*, 160561.
[8] J. Zhang, W. Yu, Y. Zhang, J. Zhu, Construction of core-shell $W_{18}O_{49}$@$ZnIn_2S_4$ hollow hierarchical structure for boosted photothermal-assisted photocatalytic H_2 production. *Int. J. Hydrogen Energy* **2024**, *85*, 165-174.
[9] X. Sun, J. Zhang, M. Luo, J. Ma, T. Xian, G. Liu, H. Yang, Elevating photocatalytic H_2 evolution over $ZnIn_2S_4$@Au@$Cd_{0.7}Zn_{0.3}S$ multilayer nanotubes via Au-mediating H–S antibonding-orbital occupancy. *Chem. Eng. J.* **2024**, *499*, 156455.
[10] P. Shan, K. Geng, Y. Shen, P. Hao, S. Zhang, J. Hou, J. Lu, F. Guo, C. Li, W. Shi, Facile synthesis of hierarchical core-shell carbon@$ZnIn_2S_4$ composite for boosted photothermal-assisted photocatalytic H_2 production. *J. Colloid Interface Sci.* **2025**, *677*, 1098-1107.
[11] Q. Li, Q. Lu, E. Guo, M. Wei, Y. Pang, Hierarchical Co_9S_8/$ZnIn_2S_4$ Nanoflower Enables Enhanced Hydrogen Evolution Photocatalysis. *Energy Fuels* **2022**, *36*, 4541-4548.
[12] R. Liu, Y. Cui, T. Wang, J. Liu, B. Liu, S. Zuo, H. Yu, A localized surface plasmon resonance effect boosts photocatalytic hydrogen evolution of $ZnIn_2S_4$/amorphous MoO_{3-x} nanodot Z-scheme heterojunctions. *J. Mater. Chem. A* **2025**, *13*, 8144-8156.
[13] W.-N. Yang, J. Yang, H. Yang, L. Sun, H.-X. Li, D.-C. Li, J.-M. Dou, X.-G. Li, G.-D. Cao, In-situ construction of tubular core–shell noble-metal-free CMT@TiO_2/$ZnIn_2S_4$ S-scheme heterojunction for superior photothermal-photocatalytic hydrogen evolution. *Rare Met.* **2025**, *44*, 2474-2488.
[14] J. Jia, X. Guo, Y. Tang, W. Zeng, H. Zeng, Z. Rui, $ZnIn_2S_4$ supported carbon dots/Ni_3P hybrid for efficient photothermal catalytic hydrogen production from water. *Int. J. Hydrogen Energy* **2025**, *104*, 122-130.
[15] X. Liu, S. Wang, J. Cao, J. Yu, J. Dong, Y. Zhao, F. Zhao, D. Zhang, X. Pu,

Anchoring $ZnIn_2S_4$ nanosheets on cross-like $FeSe_2$ to construct photothermal-enhanced S-scheme heterojunction for photocatalytic H_2 evolution. *J. Colloid Interface Sci*. **2024**, *673*, 463-474.
